# Longitudinal analysis of premotor anthropometric and serological markers of Parkinson’s disease

**DOI:** 10.1038/s41598-020-77415-1

**Published:** 2020-11-25

**Authors:** Katsunori Yokoi, Makoto Hattori, Yuki Satake, Yasuhiro Tanaka, Maki Sato, Atsushi Hashizume, Akihiro Hori, Motoshi Kawashima, Akihiro Hirakawa, Hirohisa Watanabe, Masahisa Katsuno

**Affiliations:** 1 Department of Neurology, Nagoya University Graduate School of Medicine, 65 Tsurumai-cho, Showa-ku, Nagoya, 466-8550 Japan; 2 Kumiai Kosei Hospital, Takayama, Gifu 5068502 Japan; 3 Medical Examination Centre, Daido Clinic, Nagoya, 4578511 Japan; 4 Department of Clinical Biostatistics, Graduate School of Medical and Dental Sciences, Tokyo Medical and Dental University, Tokyo, 1138510 Japan; 5 Brain & Mind Research Centre, Nagoya University Graduate School of Medicine, Nagoya, 4668560 Japan; 6 Department of Neurology, Fujita Medical University, Toyoake, Aichi 4701192 Japan

**Keywords:** Parkinson's disease, Predictive markers

## Abstract

Parkinson’s disease (PD) is a debilitating neurodegenerative disorder in which nonmotor symptoms, such as constipation and hyposmia, precede the onset of motor symptoms by 20 years. The aim of this study was to identify biomarkers at the premotor stage of PD. We assessed the differences in longitudinal changes in anthropometric and serological indices obtained from health check-up data before and after the onset of motor symptoms between male and female PD patients and healthy subjects. We enrolled 22 male and 23 female PD patients and 60 male and 60 female healthy controls. A mixed-effects model was used to estimate the trajectory of each clinical marker over the years before and after motor symptoms onset in the PD subjects, which were then compared with the trajectories of the healthy controls. The results showed a premotor blood pressure increase in female PD patients and premotor decreases in haematocrit, total cholesterol and low-density lipoprotein cholesterol in the male patients. Our results indicated that blood pressure, haematocrit and serum cholesterol levels are potential premotor markers of PD. Additionally, the changes in anthropometric and serological indices before PD motor symptoms onset were sex specific.

## Introduction

Parkinson’s disease (PD) is the second most common neurodegenerative disorder after Alzheimer’s disease. The frequency of the disorder is relatively high at 1.3 cases per 10,0000 people < 45 years old, 3100 per 10,0000 in those 75–85 years old and 4300 per 10,0000 in those > 85 years old^1,2^. PD is characterised by motor signs of bradykinesia, rigidity and resting tremor, and by nonmotor symptoms, including cognitive, neuropsychiatric, sleep, autonomic and sensory disturbances^3,4^. Some nonmotor symptoms, such as constipation, rapid eye movement sleep behaviour disorder (RBD), hyposmia and depression, are known as premotor/prodromal symptoms^5,6^. Because these symptoms manifest decades before motor symptoms, the pathogenesis of PD is thought to emerge long before the onset of motor symptoms. This view is supported by the histopathological observation that > 50% of dopaminergic neurons are lost at the onset of motor symptoms^7^.

In neurodegenerative diseases, including PD, attempts to identify biological markers for the preclinical stages have received increased attention because several studies have identified potential preclinical biomarkers. For instance, serum neurofilament light-chain levels are elevated in the preclinical stage of Alzheimer’s disease (AD) and amyotrophic lateral sclerosis/frontotemporal lobar degeneration^8–10^. The cerebrospinal fluid levels of amyloid beta and tau are established preclinical biomarkers for AD^11^. Dipeptide repeat protein is another potential preclinical marker for *C9ORF72*-associated amyotrophic lateral sclerosis^12^. Neuroimaging and neurophysiological alteration have also been detected in the preclinical phase of spinocerebellar ataxias and Huntington’s disease^13,14^.

The mean height of young adults is significantly lower in those affected by PD than in controls^15^. Weight loss in PD patients has appeared to be a continuous process that starts several years before the diagnosis of PD^16^. Anaemia is associated with PD and may precede the onset of motor symptoms by ≥ 20 years^17^. Higher serum levels of total and low-density lipoprotein (LDL) cholesterol (T-Cho and LDL-Cho, respectively) have been shown to be associated with a decreased risk of PD^18,19^, and higher serum levels of urate were also associated with a decreased risk of developing PD^20,21^.

However, little is known about the longitudinal changes in biomarkers at a premotor stage of PD. Recently, by analysing patients’ health check-up longitudinal data, we found that serum creatinine (Cr) levels started to decrease before the onset of neurological symptoms in spinal and bulbar muscular atrophy, a hereditary neuromuscular disease^22^. The aim of the present study was to examine longitudinal changes in biochemical and anthropometric indices at the premotor stage of PD. To this end, we investigated changes before and after motor symptoms onset in PD patients by analysing health check-up longitudinal data.

## Results

### Participant characteristics

**Figure 1:**
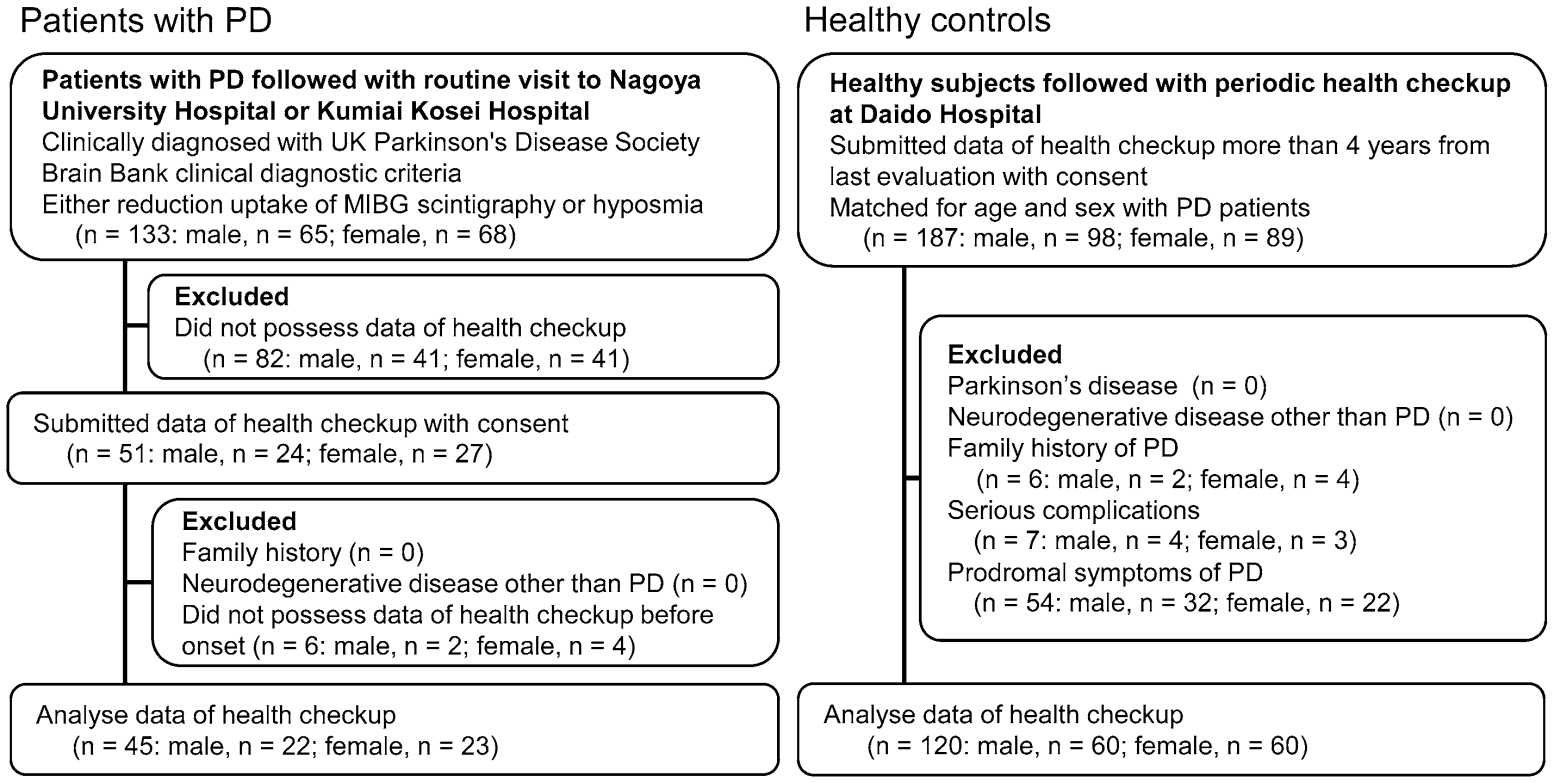
Flowchart of the study population. PD = Parkinson’s disease, MIBG = metaiodobenzylguanidine.

We recruited a total of 133 patients with PD and 187 age- and sex-matched healthy controls between October 2016 and January 2019 (Fig. 1). We excluded 88 PD patients for whom health check-up data before the onset of motor symptoms were not available; accordingly, we analysed 45 PD patients (22 males and 23 females). All included patients submitted their health checkup data > 4 years from their last evaluation and provided written informed consent. Among the potential healthy controls, we excluded six subjects who had a family history of PD, seven subjects who had serious complications (active cancer, severe anaemia, pancreatitis, epicarditis, inflammatory bowel disease, gastric ulcer or stroke) and 54 individuals who had prodromal symptoms of PD (any of hyposmia, RBD and constipation). Finally, we analysed 120 healthy controls, 60 males and 60 females. There were no significant differences in the mean ages at PD onset between the males and females (Supplemental Fig. 1). The evaluation period in all patients with PD included their premotor period. The profiles of medication history were similar between the PD subjects and healthy controls for males and females (Table 1).

**Table 1:** Clinical backgrounds of the subjects.

	Male patients with PD (n = 22)	Male healthy controls (n = 60)	*p* value
Age at motor symptoms onset (PD patients), y	65.5 ± 8.9 (48–81)	NA	0.393
Age at final evaluation (healthy controls), y	NA	64.8 ± 7.8 (47–81)	
Total evaluation period, y	13.5 ± 6.4 (5–31)	5.7 ± 0.5 (4–7)	
Initial evaluation from onset, y	7.9 ± 5.1 (2–20)	NA	
Last evaluation from onset, y	4.6 ± 3.5 (0–13)	NA	
**Medications, n (%)**			
Antihypertensive drug	3 (13.6)	14 (23.3)	0.539
Antidiabetic drug	3 (13.6)	1 (1.7)	0.057
Anticholesterolemic drug	3 (13.6)	5 (8.3)	0.437
Antihyperuricemia drug	0 (0)	1 (1.7)	1.000

	Female patients with PD (n = 23)	Female healthy controls (n = 60)	*p* value
Age at motor symptoms onset (PD patients), y	67.3 ± 7.2 (51–80)	NA	0.113
Age at the final evaluation (healthy controls), y	NA	62.8 ± 7.5 (51–74)	
Total evaluation period, y	10.8 ± 5.1 (4–21)	6.1 ± 1.1 (4–9)	
Initial evaluation from onset, y	6.9 ± 4.8 (1–17)	NA	
Last evaluation from onset, y	2.9 ± 3.5 (0–10)	NA	
**Medications, n (%)**			
Antihypertensive drug	5 (21.7 )	6 (10.0 )	0.168
Antidiabetic drug	0 (0)	1 (1.7)	1.000
Anticholesterolemic drug	4 (17.4 )	3 (5)	0.089
Antihyperuricemia drug	0 (0)	0 (0)	1.000

Data represent the mean ± standard deviation.

*PD* Parkinson’s disease, *NA* not available.

### Comparison of baseline data

First, we compared the baseline values of each item between the PD and healthy subjects for each sex separately. We defined the baseline as the onset of motor symptoms for PD groups and as the last evaluation for the healthy subjects. In males, there were significant differences between the PD patients and healthy controls in weight, body mass index (BMI), haematocrit (Ht), T-Cho, LDL-Cho and creatinine (Cr), and all the values of all these indices were lower in male PD patients than in the male healthy controls. In females, significant differences were found in height, systolic blood pressure (BP), diastolic BP, aspartate aminotransferase (AST) and T-Cho between the groups. The values of AST, systolic BP and diastolic BP were higher in female PD patients, whereas the values of other indices were lower in female PD patients than in the female healthy controls (Table 2). There were no significant differences in the clinical backgrounds, such as disease severity and medication, between the male and female patients with PD (Supplemental Table 1).

**Table 2:** Comparison of baseline data between PD patients and healthy controls.

	PD patients	Healthy controls	PD/HC (%)	*p* value
**Male**				
Height	167.0 ± 7.0 (153.1–180.9)	167.8 ± 6.1 (155.8–179.7)	99.5	0.614
Weight	63.6 ± 7.0 (45.9–81.3)	67.0 ± 7.9 (51.5–82.6)	94.9	0.094
BMI	22.7 ± 1.9 (19.0–26.4)	23.9 ± 2.4 (19.2–28.7)	94.7	0.026
Systolic BP	126.1 ± 13.1 (100.3–151.9)	123.5 ± 11.4 (101.0–146.0)	102.1	0.382
Diastolic BP	77.6 ± 6.8 (64.1–91.0)	78.1 ± 6.9 (64.6–91.7)	99.3	0.739
WBC	5374.9 ± 943.4 (3509.2–7240.7)	5449.3 ± 1386.8 (2718.0–8180.6)	98.6	0.817
RBC	461.2 ± 41.2 (380.0–542.5)	470.7 ± 32.2 (407.3–534.2)	98.0	0.276
Hb	14.5 ± 1.0 (12.5–16.5)	14.7 ± 0.9 (13.0–16.5)	93.4	0.295
Ht	42.2 ± 3.0 (36.3–48.0)	44.0 ± 2.6 (38.9–49.1)	98.4	0.008
Plt	20.4 ± 3.8 (13.0–27.9)	21.9 ± 5.4 (11.2–32.5)	95.9	0.249
AST	22.8 ± 4.0 (14.9–30.8)	22.6 ± 5.0 (12.7–32.4)	101.2	0.827
ALT	21.7 ± 8.2 (5.5–37.8)	22.9 ± 9.0 (5.2–40.7)	94.4	0.560
γ-GTP	44.1 ± 33.1 (− 22.0–110.2)	38.4 ± 19.2 (0.9–76.3)	114.1	0.359
T-Cho	190.7 ± 33.2 (124.9–256.5)	212.4 ± 21.9 (169.3–255.5)	89.8	0.001
TG	108.2 ± 65.7 (− 21.3–237.7)	128.3 ± 49.1 (31.5–225.0)	84.4	0.138
LDL-Cho	107.8 ± 25.9 (56.8–158.8)	122.9 ± 24.2 (75.4–170.5)	87.7	0.015
HDL-Cho	57.1 ± 11.2 (35.1–79.1)	59.7 ± 11.5 (37.0–82.4)	95.6	0.360
HbA1c	5.6 ± 0.6 (4.5–6.8)	5.8 ± 0.3 (5.2–6.3)	97.4	0.125
Cr	0.8 ± 0.1 (0.6–1.1)	0.9 ± 0.1 (0.7–1.1)	91.5	0.012
UA	5.6 ± 1.3 (3.0–8.1)	6.0 ± 0.8 (4.4–7.7)	92.4	0.063
TP	7.3 ± 0.4 (6.6–8.0)	7.2 ± 0.3 (6.6–7.8)	101.5	0.185
**Female**				
Height	151.9 ± 5.0 (142.1–161.7)	154.8 ± 6.1 (142.8–166.8)	98.1	0.045
Weight	49.8 ± 9.1 (31.9–67.8)	51.6 ± 7.0 (37.8–65.5)	96.5	0.344
BMI	22.1 ± 4.0 (14.2–30.1)	21.6 ± 3.2 (15.3–27.9)	102.3	0.560
Systolic BP	126.6 ± 12.1 (102.6–150.5)	119.7 ± 13.4 (93.3–146.1)	105.8	0.035
Diastolic BP	75.0 ± 7.35 (61.2–90.2)	71.4 ± 8.8 (53.9–88.8)	106.1	0.042
WBC	4931.1 ± 1103.5 (2738.6–7123.7)	4566.5 ± 1014.3 (2567.8–6565.3)	108.0	0.161
RBC	430.2 ± 31.0 (368.8–491.7)	437.3 ± 26.9 (384.3–490.2)	98.4	0.317
Hb	13.3 ± 0.9 (11.5–15.1)	13.5 ± 0.7 (12.2–14.8)	98.4	0.237
Ht	39.7 ± 2.8 (34.2–45.2)	40.7 ± 1.9 (37.0–44.4)	97.6	0.066
Plt	22.2 ± 4.9 (12.3–32.1)	22.2 ± 3.9 (14.6–29.9)	99.8	0.971
AST	23.6 ± 4.7 (14.3–32.9)	21.4 ± 3.4 (14.6–28.1)	110.4	0.020
ALT	19.1 ± 5.2 (8.9–29.4)	19.2 ± 5.3 (8.7–29.6)	99.8	0.981
γ-GTP	26.6 ± 17.4 (− 7.7–60.9)	24.5 ± 10.9 (3.1–46.0)	108.5	0.518
T-Cho	216.2 ± 26.9 (162.8–269.5)	234.9 ± 30.6 (174.7–295.1)	92.0	0.012
TG	94.8 ± 25.0 (45.5–144.1)	92.8 ± 24.0 (45.5–140.1)	102.2	0.736
LDL-Cho	122.6 ± 25.3 (72.6–172.7)	132.1 ± 25.0 (83.0–181.2)	92.8	0.131
HDL-Cho	68.3 ± 15.0 (38.6–97.9)	74.3 ± 15.8 (43.2–105.3)	91.9	0.124
HbA1c	5.7 ± 0.2 (5.2–6.2)	5.8 ± 0.3 (5.2–6.3)	99.2	0.491
Cr	0.6 ± 0.1 (0.5–0.8)	0.6 ± 0.1 (0.5–0.8)	100.4	0.883
UA	4.4 ± 0.9 (2.5–6.2)	4.6 ± 0.9 (2.9–6.3)	95.2	0.323
TP	7.2 ± 0.4 (6.3–8.0)	7.2 ± 0.3 (6.5–7.8)	100.0	0.972

Data represent the mean ± standard error.

*PD* Parkinson’s disease, *HC* healthy control, *BMI* body mass index, *BP* blood pressure, *WBC* white blood cell count, *RBC* red blood cell count, *Hb* haemoglobin, *Ht* haematocrit, *Plt* platelet count, *ALT* alanine aminotransferase, *AST* aspartate aminotransferase, *γ-GTP* gamma-glutamyl transpeptidase, *T-Cho* total cholesterol, *TG* triglyceride, *LDL-Cho* low-density lipoprotein cholesterol, *HDL-Cho* high-density lipoprotein cholesterol, *HbA1c* haemoglobin A1c, *Cr* creatinine, *UA* uric acid, *TP* total protein.

### Longitudinal changes in anthropometric markers

**Figure 2:**
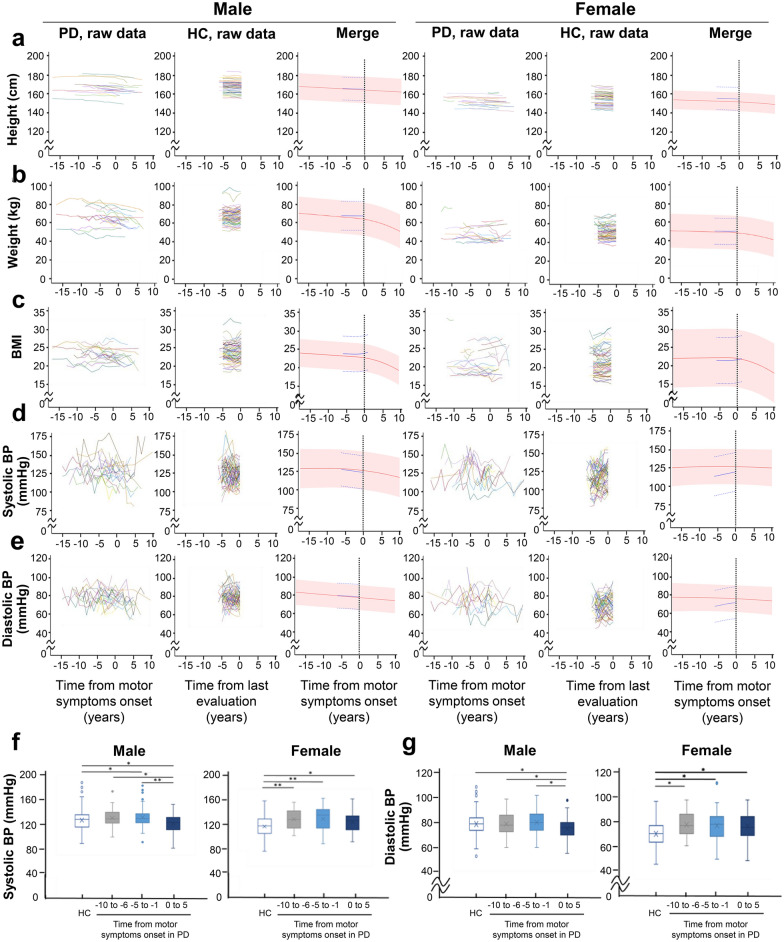
Longitudinal changes in anthropometric markers. (**a**–**e**) Raw data and estimated average trajectories of anthropometric and physical indices. Raw data for height (**a**), weight (**b**), BMI (**c**), systolic BP (**d**) and diastolic BP (**e**) are plotted against years relative to the onset of motor symptoms in patients with PD (n = 45: male, n = 22; female, n = 23) or years from the last evaluation in healthy controls (n = 120: male, n = 60; female, n = 60). In raw data graphs, each polygonal line represents longitudinal data from a single participant. The estimated average trajectory and its 95% confidence intervals estimated with a linear mixed model are plotted for each marker in patients with PD (red line and shaded area, respectively) and healthy controls (blue lines) in the merged graphs. (**f**,**g**) Box plot of anthropometric and physical indices. Values of systolic BP (**f**), diastolic BP (**g**) in each clinical stage per 5 years of PD and in HCs are shown in a plot box. *p < 0.05 and **p < 0.001. Turkey multiple comparison tests (**f**,**g**). The horizontal line in the box plot indicates the median, and the cross mark indicates the average. *PD* Parkinson’s disease, *HC* healthy control, *BMI* body mass index, *BP* blood pressure.

To detect premotor changes in indices, we analysed the estimated trajectories of the factors that showed statistically significant differences at baseline between healthy subjects and those with PD for either sex: height, weight, BMI, systolic BP and diastolic BP (Fig. 2a–e). There was no premotor change in height in either sex (Fig. 2a). The value of weight and BMI decreased after the onset of motor symptoms, but premotor alteration was not observed in both sexes (Fig. 2b,c). By contrast, the values of systolic and diastolic BP were elevated before motor onset in the female PD patients when compared with the female controls, and the difference decreased as the manifestation of motor symptoms increased, although these trends were not detected in males (Fig. 2d,e). To confirm the results of our linear mixed model analysis, we also directly compared the actual data of each item between the PD and healthy subjects for each PD disease stage (Fig. 2f,g). Increases in systolic and diastolic BP were observed > 6 years before motor onset in the female PD patients, but the differences from the control values decreased with disease progression, as shown in the estimated trajectories.

### Longitudinal changes in serological markers

**Figure 3:**
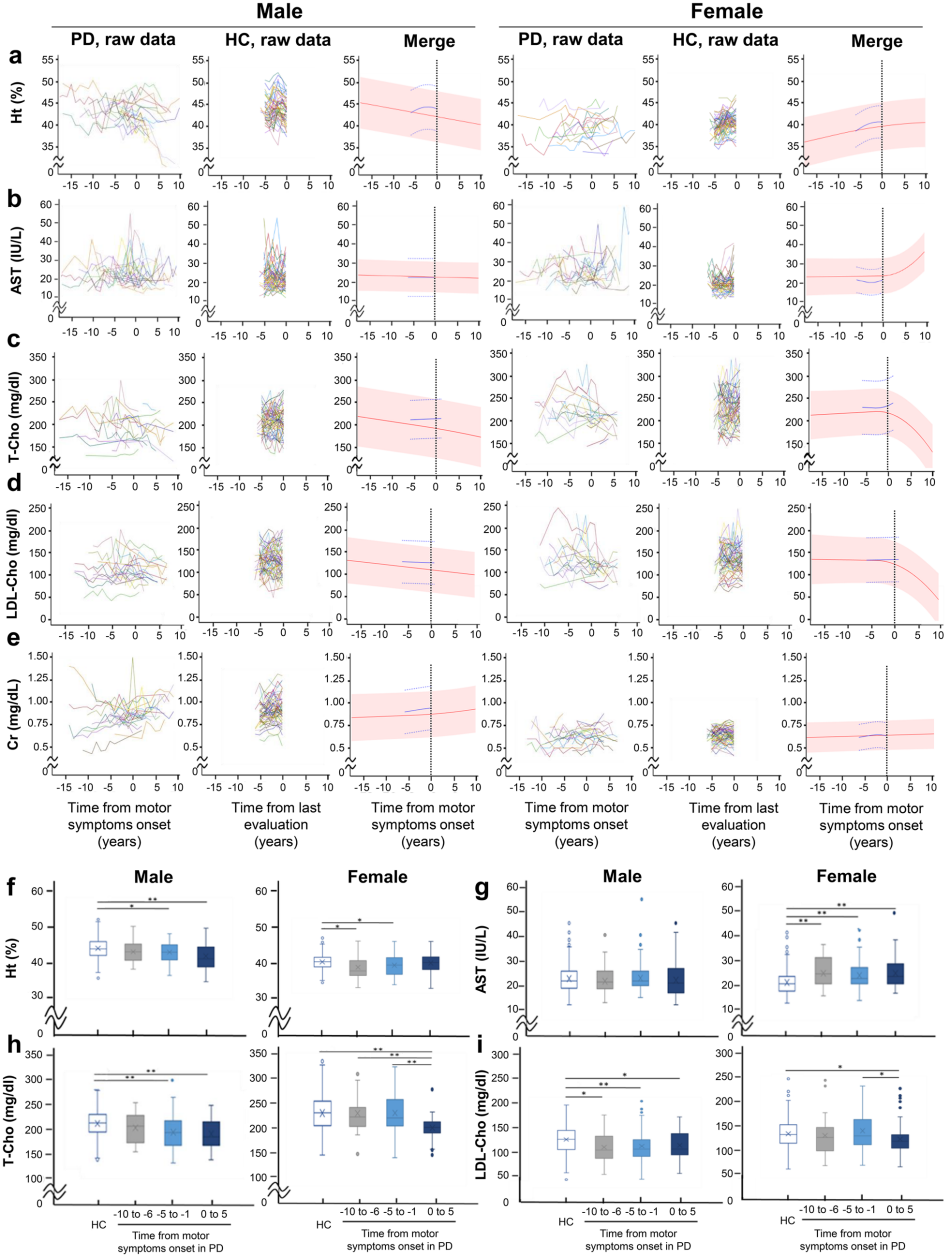
Longitudinal changes in serological markers. (**a**–**e**) Raw data and estimated average trajectories of serological indices. Raw data for Ht (**a**), AST (**b**), T-Cho (**c**), LDL-Cho (**d**) and Cr (**e**) are plotted against years relative to the onset of motor symptoms in patients with PD (n = 45: male, n = 22; female, n = 23) or years from the last evaluation in healthy controls (n = 120: male, n = 60; female, n = 60). In raw data graphs, each polygonal line represents longitudinal data from a single participant. The estimated average trajectory and its 95% confidence intervals estimated with a linear mixed model are plotted for each marker in patients with PD (red line and shaded area, respectively) and healthy controls (blue lines) in the merged graphs. (**f**–**i**) Box plot of serological indices. Values of Ht (**f**), AST (**g**), T-Cho (**h**) and LDL-Cho (**i**) in each clinical stage per 5 years of PD and in HCs are shown in a plot box. *p < 0.05 and **p < 0.001. Turkey multiple comparison tests (**f**–**i**). The horizontal line in the box plot indicates the median, and the cross mark in the box plot indicates the average. *PD* Parkinson’s disease, *HC* healthy control, *HT* haematocrit, *AST* aspartate aminotransferase, *T-Cho* total cholesterol, *LDL-Cho* low-density lipoprotein cholesterol, *Cr* creatinine.

Next, we analysed the estimated trajectories of the blood examination items, which showed statistically significant differences at baseline between PD and healthy subjects for either sex: Ht, AST, T-Cho, LDL-Cho and Cr (Fig. 3a–e). In the male PD patients, the haematocrit values started to decrease before the onset of motor symptoms (Fig. 3a). The serum values of AST increased after onset in the female PD patients, but a premotor change in this factor was not observed in either sex (Fig. 3b). The T-Cho and LDL-Cho values began to decrease before the onset of motor symptoms in the male PD patients (Fig. 3c,d). These values showed progressive declines after the onset in both sexes, but a premotor change was not observed in the female PD patients. There was no premotor change in serum Cr levels in the PD patients in either sex (Fig. 3e). In the direct comparison of actual data, the haematocrit levels in the male PD patients showed a decrease from 5 years to 1 year before the motor onset (Fig. 3f). Serum AST levels in the female PD subjects showed an increase from 10 years to 1 year before the onset of motor symptoms (Fig. 3g). Serum T-Cho levels in the male PD subjects began to decrease 5 to 1 years before the onset of motor symptoms, whereas the values decreased only after motor onset in females (Fig. 3h). Similar results were obtained for the serum LDL-Cho levels (Fig. 3i).

To exclude the influence of medications (antihypertensive agents and statins) on the longitudinal change in BP and serum lipids, we also performed subgroup analysis on the subjects not taking such drugs. In the subgroup of subjects who did not take antihypertensive agents, both systolic and diastolic BP at the premotor stage were higher in female PD patients than in the healthy controls (Supplemental Tables 2, 3, Supplemental Fig. 1). Similarly, the serum levels of T-Cho and LDL-Cho showed a premotor decline in the male statin-free subjects with PD, as observed in the analysis of the total population (Supplemental Tables 4, 5 and Supplemental Fig. 2).

Given that previous reports have suggested that low haemoglobin (Hb) and uric acid (UA) levels are potential risk factors of PD^20,21,23^, we also investigated longitudinal changes in these factors. Both Hb and UA values decreased after the onset of motor symptoms in the male PD subjects, but a change at the premotor stage was not observed (Supplemental Fig. 3A,B). There were no detectable longitudinal differences in Hb or UA between the female PD and healthy controls (Supplemental Fig. 3A,B).

## Discussion

In this study, our analysis using a linear mixed model detected an increase in BP in the premotor phase in the female PD patients. We also found that Ht decreased before motor symptoms onset in male PD patients. Furthermore, there was a premotor decline in serum T-Cho and LDL-Cho levels in the male patients, whereas these values decreased after motor symptoms onset in the female patients.

Additionally, the systolic and diastolic BP values in the female PD subjects were high but within normal range before motor symptoms onset, but there was no difference in the systolic and diastolic BP values in the males. Previous reports have suggested that systolic BP was negatively associated with PD risk in both sexes^24^. Other studies have reported that high BP in the normal range was associated with PD in women, suggesting a possible influence of oestrogen and/or autonomic dysfunction^25,26^. Given that oestrogen has been implicated in both hypertension^27,28^ and PD pathogenesis^29,30^, hormonal factors may underlie the sex-specific alteration of BP in the premotor phase of PD.

Our study showed that Ht values decreased before the motor symptoms onset in male PD patients, but Hb values showed no change. The biological indices related to anaemia have previously been shown to be risk factors of PD in various reports^23^. For example, a population-based cohort study showed that subjects with anaemia, especially with iron deficiency, were more likely to develop PD than were non-anaemic subjects^31^. The serum Ht level reflects the volume of red blood cells relative to the total blood volume^32^. Given that subjects with non-anaemic iron deficiency have normal Hb and low Ht and ferritin levels^33^, our results indicated dysregulation of iron metabolism in premotor PD individuals. It is well-known that nigral iron content is increased in PD^34^. Iron deposition in the nigra occurs even in the preclinical phase of *LRRK2* and *PRKN* mutation carriers^35^. Serum ferritin is higher in postmenopausal women than in premenopausal females^36^, and the majority of the female PD patients in our study were near the menopausal age. This finding might reflect sex-specific alteration of Ht in our cohort.

Our analysis also showed that T-Cho and LDL-Cho started to decrease before the onset of motor symptoms in the males and after onset in the females. Previous reports have stated that higher levels of T-Cho or LDL-Cho were associated with a decreased risk of developing PD, particularly in males^18^. In support of this observation, our previous study demonstrated that T-Cho levels decreased in male, but not female, at-risk PD subjects who had multiple prodromal symptoms but no motor/cognitive deficits^37^. Dysregulation of Cho could affect the nervous system in multiple ways, and Cho dysregulation has been reported in a variety of neurodegenerative diseases^38^. Lipid metabolism abnormalities have been considered a causative factor of PD^39^, and high intake of dietary Cho is likely protective against PD pathogenesis^40^. Although the mechanism underlying the association between Cho and PD is not yet known, intestinal absorption of Cho is possibly inhibited by gastrointestinal dysfunction caused by α-synuclein deposition before motor symptoms onset^41^. Accelerated lipoprotein metabolism due to oestrogen or apolipoprotein E phenotype is potentially attributable to sex-specific alteration in Cho in pre-motor PD^42,43^.

Our study had several limitations. This was a retrospective study that only included PD patients who had past health examination data before motor symptoms onset and who consented to submit their data for this study. Second, our sample size was small and limited by region and race, and items that were not included in routine health check-ups were not analysed. Third, since we selected the best fit model from among the several candidate models, we could not exclude the influence of multiplicity on our results. Consequently, we could not exclude selection bias completely, but analysing health examination data to investigate the changes in biological markers before motor symptoms onset could be generally applied to preclinical studies of other neurodegenerative analyses. Prospective studies with a larger sample size should be conducted in the future.

## Conclusions

We found that BP, Ht and serum Cho levels were potential premotor biomarkers of PD. The changes in anthropometric and serological indices before motor symptoms onset in PD subjects were different between males and females.

## Materials and methods

### Ethics

This study was conducted in accordance with the Declaration of Helsinki, the Ethics Guidelines for Human Genome/Gene Analysis Research and the Ethical Guidelines for Medical and Health Research Involving Human Subjects endorsed by the Japanese government. The study protocol was approved by the Ethics Review Committee of Nagoya University Graduate School of Medicine. We obtained written informed consent from all participants before inclusion in this study.

### Participants

PD patients were recruited from Kumiai Kosei Hospital and Nagoya University Hospital. The principal inclusion criteria were those of the UK Parkinson’s Disease Society Brain Bank clinical diagnostic criteria^44^ and either reduction uptake in ^123^I-metaiodobenzylguanidine cardiac scintigraphy or hyposmia confirmed by the odour stick identification test for Japanese^45^. We excluded PD patients for whom health checkup data before motor symptoms onset were not available, those who had a family history of PD and those who had a neurodegenerative disease other than PD.

We also recruited healthy controls from health checkup centres at Daido Hospital; they had all submitted health checkup data for > 4 years from the last evaluation and provided consent. To compare the results of the health checkup data of the controls with those of the PD patients who had not noticed motor symptoms, we matched the last evaluation age of the healthy controls with the age of motor symptoms onset of the PD patients. We excluded subjects with PD, with neurodegenerative disease other than PD, with a family history of PD, with serious complications and with prodromal symptoms of PD from the healthy controls. We used the self-reported questionnaires for exploring prodromal symptoms of PD in health checkup examinees^37^.

### Data acquisition

We acquired and assessed longitudinal data from periodic health check-ups for all patients and controls. In Japan, the law requires that employees must get a health examination that includes biochemical and anthropometric examinations once every year. Moreover, some non-employees spontaneously undergo health check-ups every year, which is why some people have their own longitudinal data either at hospitals or their homes.

The following biochemical parameters are measured commonly at cooperating facilities: height, body weight, BMI, systolic BP, diastolic BP, white blood cell count, Hb, Ht, platelet count, AST, alanine aminotransferase, gamma-glutamyl transpeptidase, T-Cho, triglycerides, LDL-Cho, high-density lipoprotein cholesterol, haemoglobin A1c (HbA1c), Cr, UA and total protein. The HbA1c level was estimated as the National Glycohaemoglobin Standardisation Programme-equivalent value (percent). BMI was calculated from the obtained height and body weight values.

In patients with PD, we analysed data obtained both before diagnosis and during follow-up after diagnosis. In the healthy controls, we analysed data obtained before the last evaluation. We evaluated all data by determining the differences in the clinical features of PD between males and females^46,47^.

### Statistical analysis

We analysed all data for each sex separately. By using a mixed-effects model, including random intercepts, we estimated average trajectories for each biomarker across years^48^. The model included polynomial basis functions to incorporate quadratic smoothing. Estimated values and 95% confidence intervals were computed from –17 to + 13 years relative to symptom onset at 1-year intervals in the patients with PD. For each index, we selected the best fit model among the several candidate models based on the goodness of fit evaluation. Multiple comparisons using statistical testing were not performed when selecting the best fitted model. Student’s *t*-test (or the Mann–Whitney *U*-test) was performed to compare the distribution of continuous variable between the two groups. The chi-squared test was used to compare the distribution of categorical variables between the two groups. Pearson correlation coefficients were used to analyse correlations among parameters. Continuous and categorical data are presented as the mean ± SD and frequency (proportion), respectively, unless stated otherwise. We considered *p* < 0.05 to be indicative of statistical significance. The Statistical Package for the Social Sciences 25.0 J software (IBM Japan, Tokyo, Japan) and SAS version 9.4 (SAS Institute, Inc., Cary, NC, USA) were used to perform all statistical analyses.

## Supplementary information

Supplementary Information

## Data Availability

The datasets generated during and/or analysed during the current study are available from the corresponding author on reasonable request.
