# Supplementary material for: Longitudinal analysis of premotor anthropometric and serological markers of Parkinson’s disease
Source: Sci Rep. 2020 Nov 25;10:20524. doi: 10.1038/s41598-020-77415-1 (PMC7688961; doi:10.1038/s41598-020-77415-1)
Supplement: Supplementary file 1 — Supplementary Information [file 41598_2020_77415_MOESM1_ESM.docx]

**Supplementary information**

**Longitudinal analysis of premotor anthropometric and serological markers of Parkinson’s disease**

Katsunori Yokoi, MD, Makoto Hattori, MD, Yuki Satake, MD, Yasuhiro Tanaka, PhD, Maki Sato, Atsushi Hashizume, MD, PhD, Akihiro Hirakawa, PhD, Akihiro Hori, MD, Motoshi Kawashima, MD, Hirohisa Watanabe, MD, PhD and Masahisa Katsuno, MD, PhD.

**Contents:**

Four Supplemental Figures (Supplemental Figures 1—3)

Four Supplemental Tables (Supplemental Tables 1—5)

**Supplemental Figure 1. Raw data and estimated average trajectories of blood pressure excluding antihypertensive drug users**

**
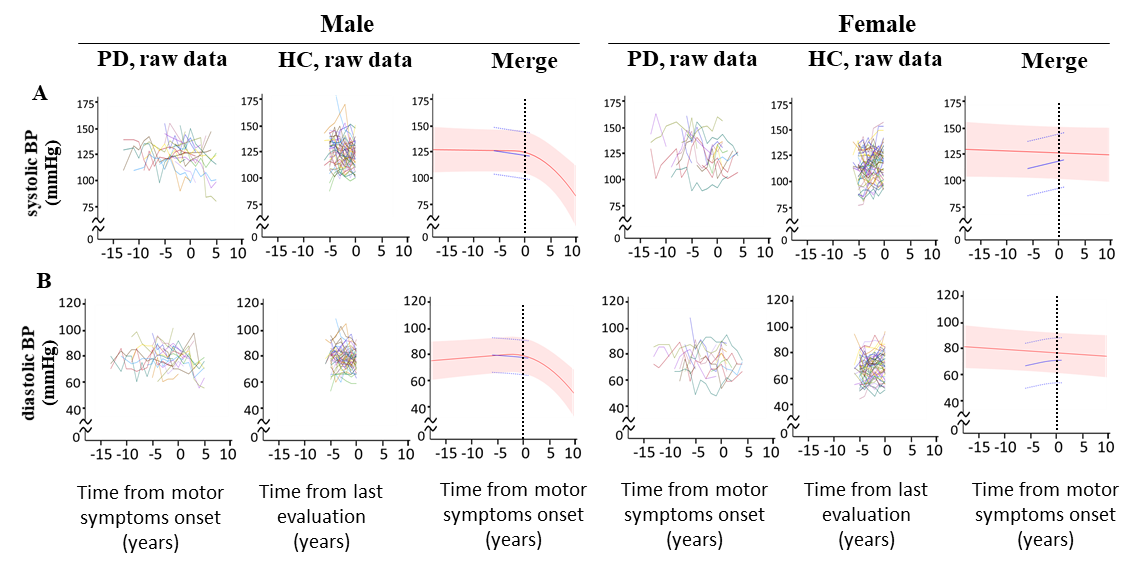
**

Raw data for systolic BP (A), diastolic BP (B) and systolic BP (excluding BP of antihypertensive agent users) (C) and diastolic BP (excluding BP of antihypertensive agent users) (D) are plotted against years relative to the onset of motor symptoms in patients with PD (n = 45: male, n = 22; female, n = 23) or years from the last evaluation in healthy controls (n = 120: male, n = 60; female, n = 60). In raw data graphs, each polygonal line represents longitudinal data from a single participant. The estimated average trajectory and its 95% confidence intervals estimated with a linear mixed model are plotted for each marker in patients with PD (red line and shaded area, respectively) and healthy controls (blue lines) in the merged graphs.

PD = Parkinson’s disease, HC = healthy control, BP = blood pressure

**Supplemental Figure 2. Raw data and estimated average trajectories of cholesterol, excluding statin users**

**
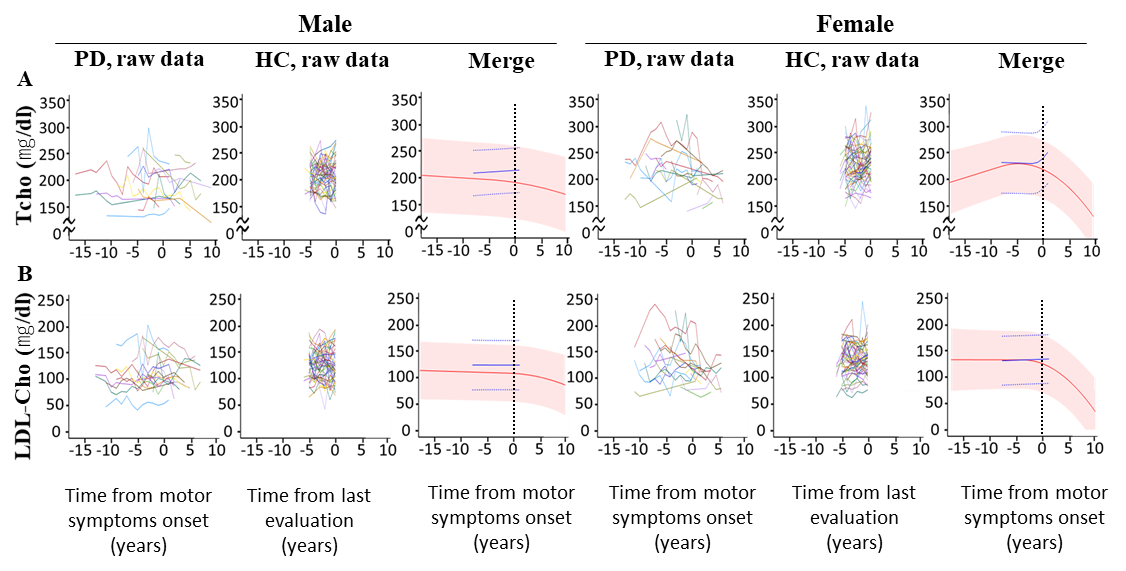
**

Raw data for T-Cho (A), LDL-Cho (B), and T-Cho (excluding statin users) (C), LDL-Cho (excluding statin users) (D) are plotted against years relative to the onset of motor symptoms in patients with PD (n = 45: male, n = 22; female, n = 23) or years from the last evaluation in healthy controls (n = 120: male, n = 60; female, n = 60). In raw data graphs, each polygonal line represents longitudinal data from a single participant. The estimated average trajectory and its 95% confidence intervals estimated with a linear mixed model are plotted for each marker in patients with PD (red line and shaded area, respectively) and healthy controls (blue lines) in the merged graphs.

PD = Parkinson’s disease, HC = healthy control, T-Cho = total cholesterol, LDL-Cho = low-density lipoprotein cholesterol

**Supplemental Figure 3. Raw data and estimated average trajectories of other biomarkers**


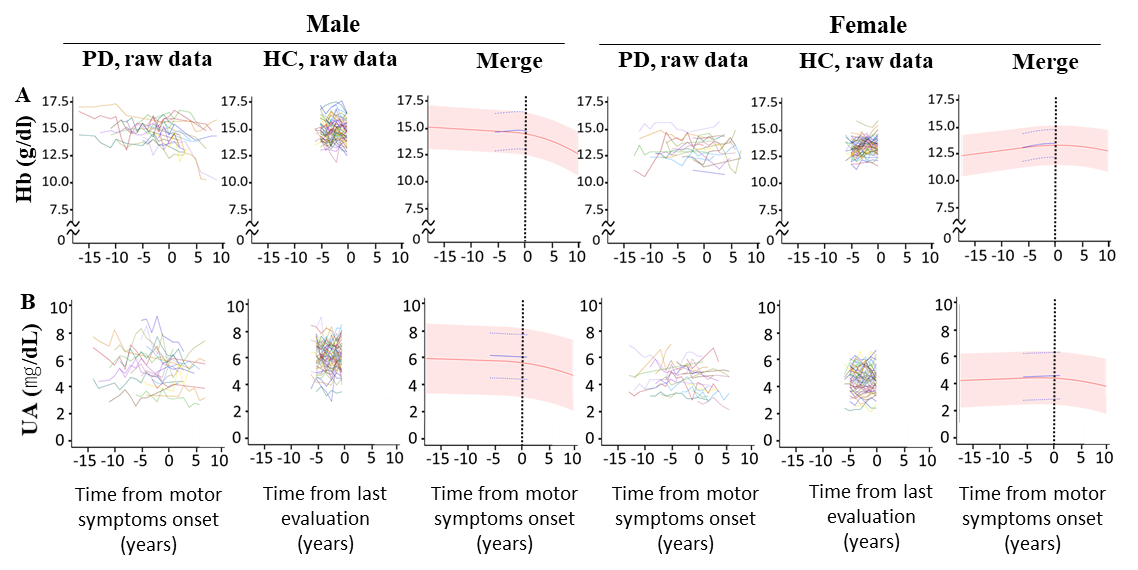


Raw data for Hb (A), UA (B) are plotted against years relative to the onset of motor symptoms in patients with PD (n = 45: male, n = 22; female, n = 23) or years from the last evaluation in healthy controls (n = 120: male, n = 60; female, n = 60). In raw data graphs, each polygonal line represents longitudinal data from a single participant. The estimated average trajectory and its 95% confidence intervals estimated with a linear mixed model are plotted for each marker in patients with PD (red line and shaded area, respectively) and healthy controls (blue lines) in the merged graphs.

PD = Parkinson’s disease, HC = healthy control, Hb = haemoglobin, UA = uric acid

**Supplemental Table 1. Clinical backgrounds of male and female Parkinson’s disease patients**

|  | **Male patients with PD (n = 22)** | **Female patients with PD (n = 23)** | ***p* value** |
| --- | --- | --- | --- |
| **Age at motor symptoms onset, y** | **65.5 ± 9.2 (51–80)** | **67.3 ± 7.2 (51–80)** | **0.459** |
| **Total evaluation period, y** | **13.5 ± 6.4 (5–31)** | **10.8 ± 5.1 (4–21)** | **0.135** |
| **Initial evaluation from onset, y** | **7.9 ± 5.1 (2–10)** | **6.9 ± 4.8 (1–17)** | **0.516** |
| **Last evaluation from onset, y** | **4.5 ± 3.5 (0–13)** | **2.9 ± 3.5 (0–10)** | **0.127** |
| **Hoehn-Yahr scale of Parkinson's disease** | **2.4 ± 0.9 (1–4)** | **1.9 ± 0.9 (1–4)** | **0.076** |
| **UPDRS III** | **24.2 ± 8.4 (14–42)** | **20.2 ± 7.5 (5–33)** | **0.185** |
| **UPDRS Total** | **46.2 ± 11.5 (33–72)** | **36.6 ± 17.5 (8–73)** | **0.109** |
| **LEDD** | **394.2 ± 286.9 (25–1280)** | **363.4 ± 248.3 (80–1138)** | **0.715** |
| **MoCA-J** | **23.5 ± 5.3 (6–28)** | **25.6 ± 3.3 (19–30)** | **0.208** |

Data represent mean ± standard deviation

PD = Parkinson’s disease, y = years, UPDRS = unified Parkinson's disease rating scale, LEDD = L-dopa equivalent daily dose, MoCA-J = Japanese version of the Montreal cognitive assessment

**Supplemental Table 2. Estimated values at onset of PD, excluding antihypertensive drug users**

|  | **Male patients with PD(n=18)** | **Male healthy controls(n=46)** | ***p* value** |
| --- | --- | --- | --- |
| **Age at motor symptoms onset (PD patients), y** | **66.8 ± 8.6 (50–81)** | **NA** | **0.176** |
| **Age at the final evaluation (healthy controls), y** | **NA** | **63.7 ± 7.9 (47–81)** |  |
| **Total evaluation period, y** | **12.5 ± 5.4 (5–29)** | **5.4 ± 1.7 (1–6)** |  |
| **Initial evaluation from onset, y** | **7.2 ± 5.0 (2–20)** | **NA** |  |
| **Last evaluation from onset, y** | **4.3 ± 3.2 (0–12)** | **NA** |  |
| **Medications, n (%)** |  |  |  |
| **Antihypertensive drug** | **0 (0)** | **0 (0)** | **0.189** |
| **Antidiabetic drug** | **2 (11.1)** | **1 (2.2)** | **0.057** |
| **Anticholesterolemic drug** | **1 (5.6)** | **3 (6.5)** | **1.000** |
| **Antihyperuricemia drug** | **0 (0)** | **0 (0)** | **1.000** |
|  | **Female patients with PD(n=18)** | **Female healthy controls(n=54)** | ***p* value** |
| **Age at motor symptoms onset (PD patients), y** | **68.0 ± 6.5 (57–80)** | **NA** | **0.009** |
| **Age at the final evaluation (healthy controls), y** | **NA** | **63.4 ± 5.7 (51–72)** |  |
| **Total evaluation period, y** | **10.7 ± 5.6 (3–21)** | **6.0 ± 0.6 (4–8)** |  |
| **Initial evaluation from onset, y** | **6.9 ± 5.1 (0–17)** | **NA** |  |
| **Last evaluation from onset, y** | **3.1 ± 3.6 (0–10)** | **NA** |  |
| **Medications, n (%)** |  |  |  |
| **Antihypertensive drug** | **0 (0 )** | **0 (0 )** | **1.000** |
| **Antidiabetic drug** | **0 (0)** | **0 (0)** | **1.000** |
| **Anticholesterolemic drug** | **2 (11.1 )** | **3 (5.6)** | **0.593** |
| **Antihyperuricemia drug** | **0 (0)** | **0 (0)** | **1.000** |

Data represent the mean ± standard deviation

PD = Parkinson’s disease, NA = not available, y = years

**Supplemental Table 3. Estimated values at onset of PD, excluding antihypertensive drug users**

|  | **PD patients** | **Healthy controls** | ***p* value** |
| --- | --- | --- | --- |
| **Systolic BP (male)** | **125.1 ± 10.3 (104.68–145.4)** | **122.0 ± 11.5 (99.3–144.8)** | **0.283** |
| **Diastolic BP (male)** | **79.0 ± 6.9 (65.4–92.6)** | **77.5 ± 6.7 (64.3–90.8)** | **0.393** |
| **Systolic BP (female)** | **126.6 ± 12.7 (101.5–151.7)** | **118.6 ± 13.3 (92.5–144.8)** | **0.016** |
| **Diastolic BP (female)** | **76.5 ± 7.9 (60.9–92.1)** | **70.6 ± 8.9 (53.1–88.1)** | **0.007** |

Data represent the mean ± standard deviation

PD = Parkinson’s disease, HC = healthy control, BP = blood pressure

**Supplemental Table 4. Clinical background of PD patients, excluding statin users**

|  | **Male patients with PD (n=19)** | **Male healthy controls (n=55)** | ***p* value** |
| --- | --- | --- | --- |
| **Age at motor symptoms onset (PD patients), y** | **67.4 ± 7.8 (50–81)** | **NA** | **0.213** |
| **Age at the final evaluation (healthy controls), y** | **NA** | **64.6 ± 8.1 (47–81)** |  |
| **Total evaluation period, y** | **13.0 ± 5.2 (6–29)** | **5.6 ± 1.2 (4–7)** |  |
| **Initial evaluation from onset, y** | **7.6 ± 5.0 (2–20)** | **NA** |  |
| **Last evaluation from onset, y** | **4.4 ± 3.1 (0–12)** | **NA** |  |
| **Medications, n (%)** |  |  |  |
| **Antihypertensive drug** | **1 (5.2)** | **10 (17.5)** | **0.269** |
| **Antidiabetic drug** | **2 (10.5)** | **1 (1.8)** | **0.160** |
| **Anticholesterolemic drug** | **0 (0)** | **0 (0)** | **1.000** |
| **Antihyperuricemia drug** | **0 (0)** | **0 (0)** | **1.000** |
|  | **Female patients with PD (n=19)** | **Female healthy controls (n=57)** | ***p* value** |
| **Age at motor symptoms onset (PD patients), y** | **68.0 ± 7.7 (51–80)** | **NA** | **0.009** |
| **Age at the final evaluation (healthy controls), y** | **NA** | **63.7 ± 5.6 (51–74)** |  |
| **Total evaluation period, y** | **10.9 ± 5.4 (4–21)** | **5.9 ± 0.7 (3–8)** |  |
| **Initial evaluation from onset, y** | **7.4 ± 4.8 (7–17)** | **NA** |  |
| **Last evaluation from onset, y** | **2.7 ± 3.8 (0–10)** | **NA** |  |
| **Medications, n (%)** |  |  |  |
| **Antihypertensive drug** | **3 (15.8 )** | **5 (8.8 )** | **0.405** |
| **Antidiabetic drug** | **0 (0)** | **1 (1.8)** | **1.000** |
| **Anticholesterolemic drug** | **0 (0 )** | **0 (0)** | **1.000** |
| **Antihyperuricemia drug** | **0 (0)** | **0 (0)** | **1.000** |

Data represent the mean ± standard deviation

PD = Parkinson’s disease, NA = not available, y = years

**Supplemental Table 5. Estimated values at onset of PD, excluding statin users**

|  | **PD patients** | **Healthy controls** | ***p* value** |
| --- | --- | --- | --- |
| **T-Cho (male)** | **190.7 ± 34.9 (121.4–260.0)** | **213.4 ± 21.6 (170.8–256.0)** | **0.001** |
| **LDL-Cho (male)** | **108.8 ± 27.0 (55.5–162.1)** | **124.5 ± 23.7 (77.9–171.1)** | **0.011** |
| **T Cho (female)** | **216.1 ± 26.9 (162.8–269.6)** | **234.9 ± 30.6 (174.7–295.1)** | **0.011** |
| **LDL-Cho (female)** | **122.6 ± 25.3 (72.6–172.7)** | **132.1 ± 24.94 (82.7–181.2)** | **0.125** |

Data represent the mean ± standard deviation

PD = Parkinson’s disease, HC = healthy control, T-Cho = total cholesterol, LDL-Cho = low-density lipoprotein cholesterol
